# Bladder cancer cells shift rapidly and spontaneously to cisplatin-resistant oxidative phosphorylation that is trackable in real time

**DOI:** 10.1038/s41598-022-09438-9

**Published:** 2022-04-01

**Authors:** Tong Xu, Jason A. Junge, Alireza Delfarah, Yi-Tsung Lu, Cosimo Arnesano, Maheen Iqbal, Kevin Delijani, Tien-Chan Hsieh, Emmanuelle Hodara, Hemal H. Mehta, Pinchas Cohen, Nicholas A. Graham, Scott E. Fraser, Amir Goldkorn

**Affiliations:** 1 Division of Medical Oncology, Department of Internal Medicine, University of Southern California Keck School of Medicine and Norris Comprehensive Cancer Center, 1441 Eastlake Avenue, Suite 3440, Los Angeles, CA 90033 USA; 2 Translational Imaging Center, University of Southern California, Los Angeles, CA 90089 USA; 3 Mork Family Department of Chemical Engineering and Materials Science, University of Southern California, Los Angeles, CA 90089 USA; 4 Calico Life Sciences LLC, South San Francisco, Los Angeles, CA 94080 USA; 5 The Leonard Davis School of Gerontology, University of Southern California, Los Angeles, CA 90089 USA; 6 Department of Biochemistry & Molecular Medicine, University of Southern California Keck School of Medicine, Los Angeles, CA 90033 USA

**Keywords:** Cancer metabolism, Cancer stem cells, Urological cancer, Time-lapse imaging

## Abstract

Genetic mutations have long been recognized as drivers of cancer drug resistance, but recent work has defined additional non-genetic mechanisms of plasticity, wherein cancer cells assume a drug resistant phenotype marked by altered epigenetic and transcriptional states. Currently, little is known about the real-time, dynamic nature of this phenotypic shift. Using a bladder cancer model of nongenetic plasticity, we discovered that rapid transition to drug resistance entails upregulation of mitochondrial gene expression and a corresponding metabolic shift towards the tricarboxylic acid cycle and oxidative phosphorylation. Based on this distinction, we were able to track cancer cell metabolic profiles in real time using fluorescence lifetime microscopy (FLIM). We observed single cells transitioning spontaneously to an oxidative phosphorylation state over hours to days, a trend that intensified with exposure to cisplatin chemotherapy. Conversely, pharmacological inhibition of oxidative phosphorylation significantly reversed the FLIM metabolic signature and reduced cisplatin resistance. These rapid, spontaneous metabolic shifts offer a new means of tracking nongenetic cancer plasticity and forestalling the emergence of drug resistance.

## Introduction

Major strides in cancer treatment seldom result in lasting cures due to the emergence of resistance. Drug resistant tumor cell populations are traditionally conceptualized as arising by selection and clonal expansion of rare cells bearing adaptive DNA driver mutations^1–3^. However, several lines of evidence now suggest that resistance is not achieved via genetic alterations alone, but also through more fluid epigenetic, transcriptional, and metabolic mechanisms that endow cancer cells with phenotypic plasticity, the ability to shift to a drug-resistant state independently of new mutations^4–18^. Maintaining a dynamic equilibrium of multiple cell states, so-called “bet-hedging”, maximizes a tumor’s overall fitness and allows it to persist in the face of environmental stressors like drug treatment^4,5,7,12,13,19,20^. Consistent with this idea, we previously used bladder cancer cell line models to show that aggressive, drug resistant, cancer stem-like cells arise spontaneously, rapidly and repeatedly over time from isogenic cells lacking these properties, and that these phenotypic shifts are associated with changes in DNA methylation, chromatin accessibility, and downstream signaling^7,12,13^.

Studies of cancer plasticity and drug resistance have generally been hampered by the requirement for large cell populations assayed at defined time points, yielding a limited “before and after” glimpse of underlying cellular phenotypic shifts. To address this gap, we set out to identify a plasticity feature that can be measured at the single cell level and tracked non-invasively, in-situ, over time. We used our established bladder cancer cell line model^7,12,13^, wherein we previously reported nongenetic inter-conversion between two distinct phenotypes: an aggressive, drug-resistant, cancer stem-like side population (SP) of cells, and an isogenic non-side population (NSP) of cells lacking these properties (Supplemental Fig. S1). Transcriptional profiling by RNAseq revealed that the most highly upregulated genes in SP cells were genes mediating mitochondrial oxidative phosphorylation (OxPhos). These findings were validated by metabolomic and functional metabolic assays that confirmed significantly higher OxPhos in SP cells relative to NSP cells. Next, we tested whether the differential metabolic state of SP cells could be visualized using fluorescence lifetime microscopy (FLIM), which measures the cells’ free/bound NADH + redox states^21–24^. We found that SP cells have a distinct OxPhos FLIM signature, which we exploited to identify and track single cells over time as they shifted from the NSP to SP phenotype. These shifts occurred spontaneously, were accelerated by treatment with cisplatin chemotherapy and reversed by treatment with phenformin, an OxPhos inhibitor. To our knowledge, this is the first report defining a metabolic shift to a more drug-resistant OxPhos state occurring rapidly (hours) in single cancer cells. This newly identified rapid metabolic plasticity opens new avenues for tracking and analyzing cell fates in situ, and for therapeutically targeting metabolic plasticity as a means to prevent transition to an aggressive, drug resistant cancer phenotype.

## Results

**Figure 1:**
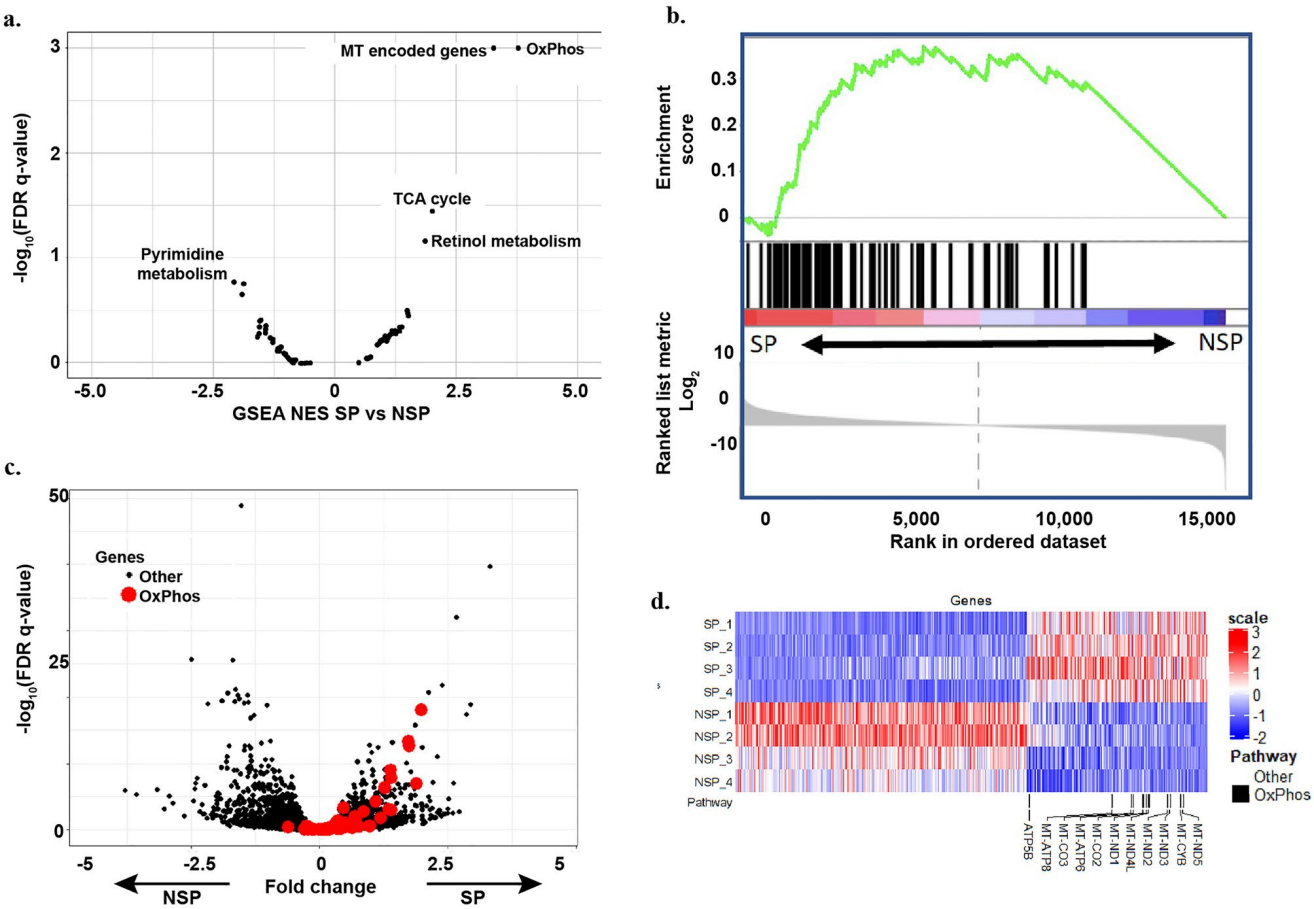
Gene expression in SP cells is enriched for OxPhos pathway. (**a**) Oxidative phosphorylation pathway was the most highly enriched in the KEGG metabolic pathways. (**b**) GSEA analysis demonstrated that the OxPhos gene set was significantly enriched in SP (normalized enrichment score (NES) 3.97, nominal *p* value < 0.0002, FDR *q* value < 0.0002). (**c**) Volcano plot of differential gene expression between SP and NSP cells. OxPhos genes (red) were predominantly overexpressed in SP as compared with the entire transcriptome (black). (**d**) Heatmap (Transcripts Per Million, TPM) of all significantly differentially expressed genes demonstrating OxPhos gene overexpression in SP. The heatmap was created using the Complex Heatmap package v.2.4.3 (PMID: 27207943) in R 4.0.1. https://bioconductor.org/packages/release/bioc/html/ComplexHeatmap.html.

SP and NSP cells were recovered by Hoechst exclusion staining and FACS from J82 and T24 bladder cancer cell lines as reported previously^7,12,13^. RNAseq and gene set enrichment analysis (GSEA)^25^ revealed that OxPhos and mitochondria-encoded gene sets were most significantly enriched in SP cells across all metabolic gene sets in the Kyoto Encyclopedia of Genes and Genomes (KEGG)^26^ (Fig. 1a, b). When all differentially expressed genes between SP and NSP were analyzed, OxPhos genes were predominantly overexpressed in SP (Fig. 1c). Furthermore, among the genes differentially expressed between SP and NSP, all the OxPhos genes were upregulated in SP (Fig. 1d).

**Figure 2:**
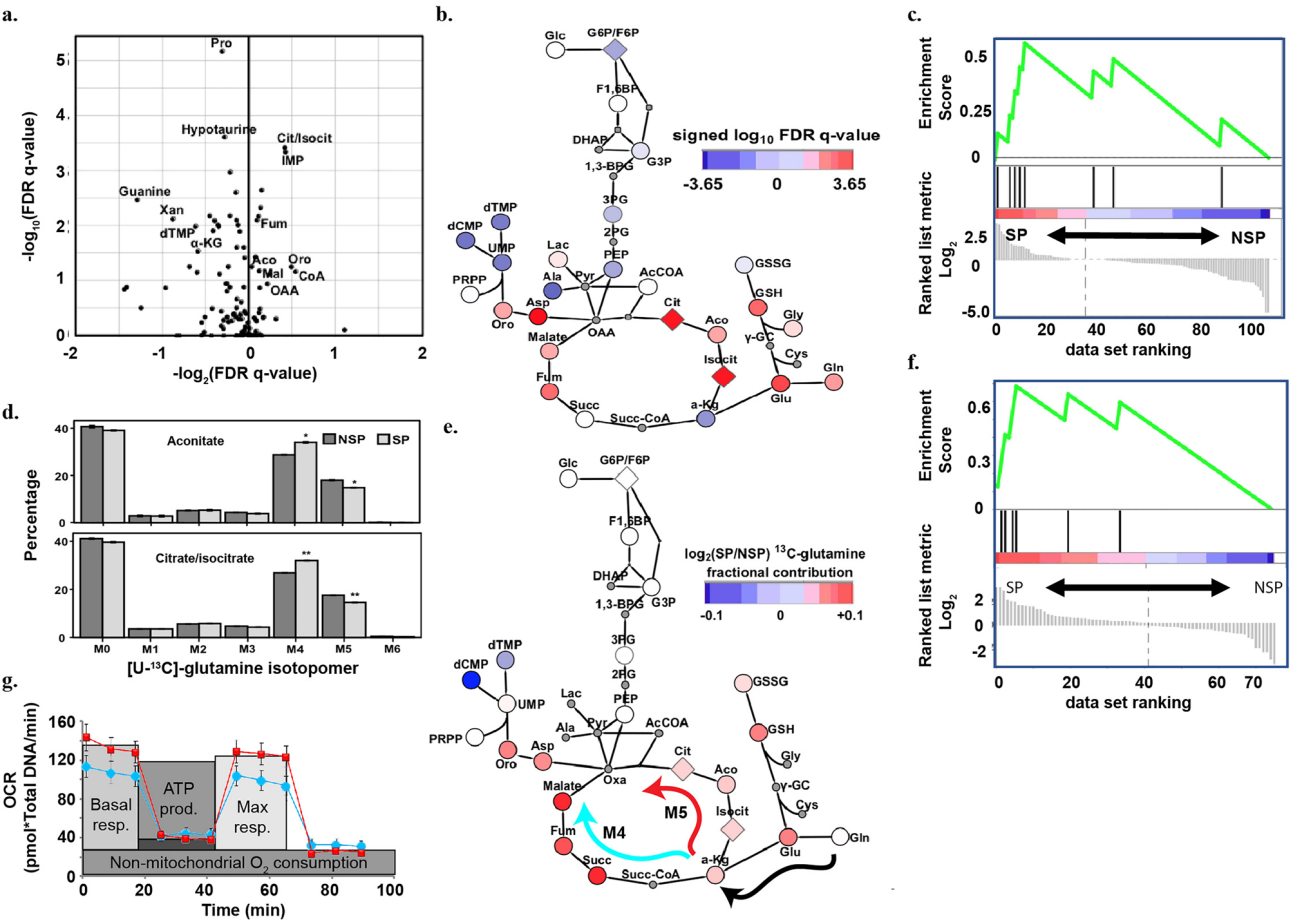
Glutamine-derived TCA cycle is upregulated in SP population. (**a**) Volcano plot of intracellular metabolite pool sizes. Data represents average weighted log_2_ fold change (SP/NSP) and FDR-corrected q-value from three independent experiments. (**b**) Metabolic pathway map representing signed log_10_ FDR q-value on a color scale for intracellular pool sizes of metabolites comparing SP and NSP populations. Metabolites that were not measured are shown as small circles with grey color. Isomers that were not resolved by LC–MS are shown as diamonds. (**c**) Enrichment of TCA cycle pathway with MSEA analysis of intracellular metabolites pool sizes of SP and NSP populations. Metabolites were ranked based on the signed log_10_ FDR q-value comparing SP and NSP populations. (**d**) Labelling patterns of aconitate and citrate/isocitrate from [U-^13^C]-glutamine. M-values reflect the number of ^13^C (5 total) remaining after α-ketoglutarate is modified in the TCA cycle. M4 is associated with conversion of α-ketoglutarate to succinyl-CoA, giving up one heavy carbon in the reaction, and is coupled to oxidative phosphorylation. M5 is associated with carboxylation of α-ketoglutarate to become isocitrate and retaining all 5 ^13^C atoms. This reversal of TCA is also known as “backwards” flux. * denotes p-value less than 0.0002, ** denotes *p* value less than 0.00004 by Student’s *t* test. Results are means of three biological replicates with small error bars; raw data are provided in Supplemental Table S1. (**e**) Metabolic pathway map representing log_2_ fold change of SP/NSP on a color scale for [U-^13^C]-glutamine fractional contribution. The arrows show the two paths, M4 forward flux (blue arrow) and M5 backward flux (red arrow) that [U-^13^C]-glutamine can take after becoming α-ketoglutarate and entering TCA cycle (black arrow). Metabolites that were not measured or had less than 1% fractional contribution are shown as small circles with grey color. Isomers that were not resolved by LC–MS are shown as diamonds. (**f**) Enrichment of TCA cycle pathway with MSEA analysis of [U-^13^C]-glutamine fractional contribution of SP and NSP populations. Metabolites were ranked by the log_2_ ratio of SP/NSP [U-^13^C]-glutamine fractional contribution. (**g**) OCR in SP (red) is higher than NSP (blue) in the Seahorse analysis corresponding to the expected higher OxPhos metabolism of SP cells. *(**b**) and (**e**) were drawn using cytoscape: version 3.5.1. https://cytoscape.org/roadmap.html.

Having identified a significant transcriptional signature associated with OxPhos in SP cells, we turned to explore the relative metabolic states of SP and NSP subpopulations. First, we profiled central carbon metabolism in SP and NSP cells by measuring intracellular metabolite pool sizes using liquid chromatography-mass spectrometry (LC–MS) based metabolomics^27^. This analysis revealed that the tricarboxylic acid (TCA) cycle metabolites citrate/isocitrate and fumarate were significantly upregulated in the SP subpopulation (Fig. 2a), as were aconitate, malate, and oxaloacetate. Visualization of the signed log_10_ FDR q-value on a metabolic pathway map showed that all TCA cycle metabolites except for alpha-ketoglutarate and succinate were upregulated in SP cells (Fig. 2b). To test whether the TCA cycle was significantly enriched relative to other pathways in SP cells, we performed metabolite set enrichment analysis (MSEA) using intracellular metabolite pool sizes. Indeed, MSEA confirmed that the TCA cycle was the most enriched pathway in the SP population (Fig. 2c). In comparison, none of the glycolytic metabolites were significantly altered between SP and NSP cells, and MSEA did not indicate an enrichment of glycolysis in SP or NSP populations.

Next, to better understand the differences in metabolic flux between SP and NSP subpopulations, we performed stable isotope tracing metabolomics. We first cultured cells with [U-^13^C]-glutamine as a tracer to differentiate between oxidative metabolism and reductive carboxylation in the TCA cycle. Specifically, oxidative TCA cycle flux results in the loss of one heavy carbon and the labeling of downstream TCA cycle metabolites with only four heavy carbons (Fig. 2d, M4), whereas reductive carboxylation retains all five heavy carbons on isocitrate, aconitate, and citrate (Fig. 2d, M5)^28^. Isotopomer distributions for metabolites from the TCA cycle revealed that SP cells exhibit an increased percentage of M4 aconitate and citrate/isocitrate, suggesting increased oxidative metabolism (Fig. 2d). SP cells also had a complementary reduction in M5 aconitate and citrate/isocitrate percentages, suggesting reduced reductive carboxylation in SP cells. Additionally, we visualized the total fractional contribution of [U-^13^C]-glutamine on a metabolic pathway map^29^ and found that SP cells exhibited an increased contribution of glutamine-derived carbon to the TCA cycle, glutathione metabolism, and aspartate (Fig. 2e). Furthermore, MSEA revealed that the fractional contribution of glutamine to TCA cycle metabolites was significantly enriched relative to other metabolic pathways (Fig. 2f). We next labeled cells with [U-^13^C]-glucose and performed LC–MS metabolomics on flow-sorted SP and NSP populations. We again observed an increased fractional contribution of [U-^13^C]-glucose to most TCA cycle metabolites in SP cells (Supplemental Fig. S2). Next, we measured the oxygen consumption rates (OCR) of SP and NSP cells using the Seahorse assay. Consistent with our metabolomic data, we found that SP cells exhibited a significantly increased rate of both basal and maximal OCR compared to NSP cells (Fig. 2g). Taken together, these data support that SP cells have increased levels of mitochondrial aerobic metabolism than NSP cells.

Having established the differential metabolic states of SP and NSP cells, we asked whether cell transitions between these states could be tracked using two-photon FLIM, a non-invasive, non-interventive and label-free microscopy method that allows real-time tracking of metabolism in cells and tissues^21,30,31^. Here, we image NADH as the intrinsic fluorescent marker and measure its fluorescence decay times (lifetime) to monitor cellular energy metabolism. A short fluorescence decay lifetime is correlated with a high ratio of bound/total NADH, which corresponds to a lower ratio of free NADH/NAD+ and a more OxPhos metabolic state^21–24,30–32^. Supplemental Fig. S3 describes decay trends and analysis of NADH FLIM data in the lifetime-phasor. The phasor’s graphical interface is an elegant and powerful tool for interacting with live-cell imaging data and for extracting meaning out of metabolic imaging.

**Figure 3:**
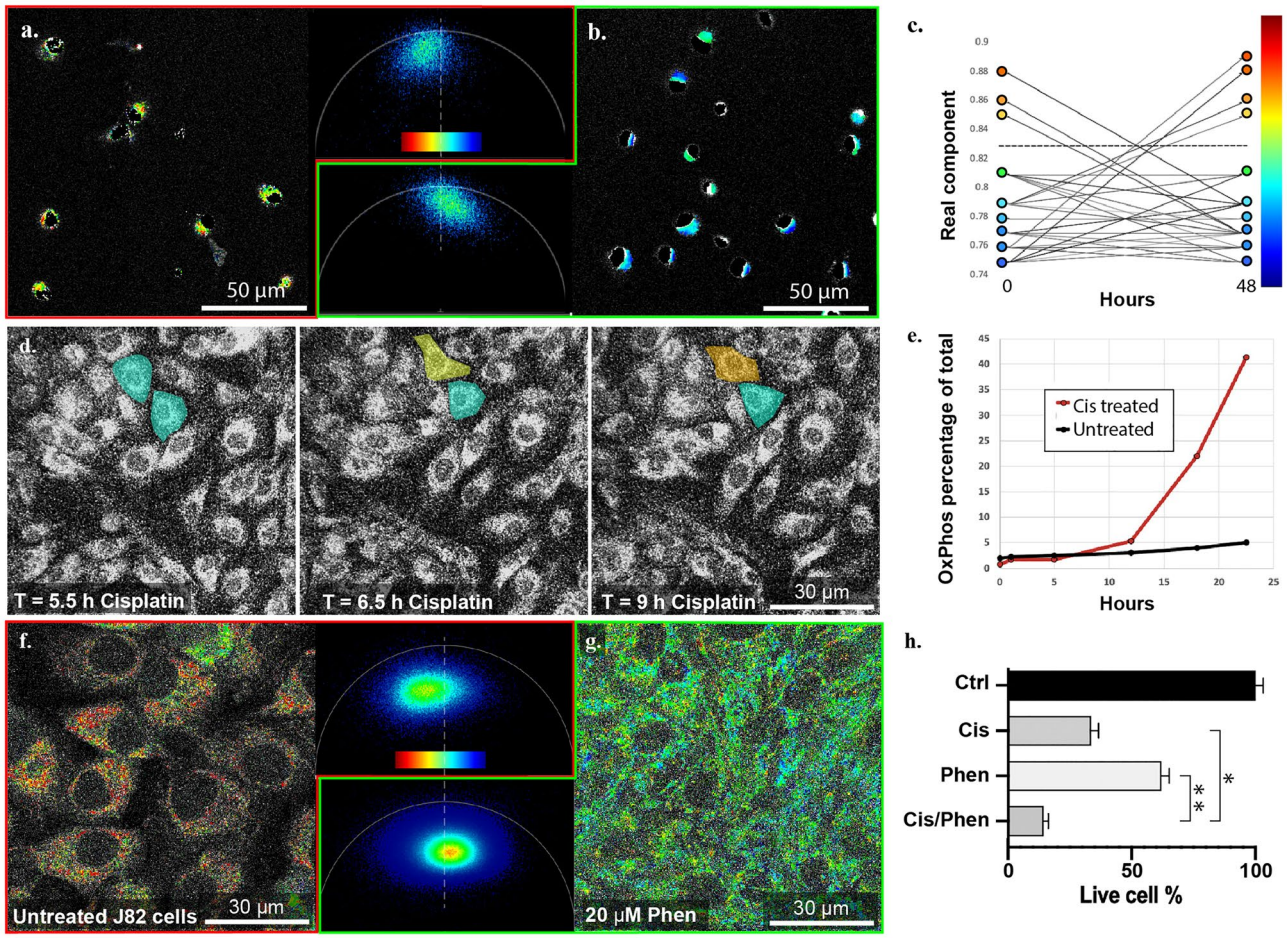
Single-cell analysis of metabolic states. (**a**, **b**) J82 cells were sorted into SP and NSP cultures by FACS and analyzed in FLIM phasors. SP cells (**a**) have a phasor distribution that describes more OxPhos metabolic states whereas NSP cells (**b**) have a phasor distribution that describes more glycolytic metabolic. The dashed line in the phasor panels of a and b represents an identifiable boundary between NSP and SP FLIM signals, and the color scale bar applies to the cell images of (**a**) and (**b**). (**c**) In unsorted J82 cell culture, we tracked the metabolism of 35 cells for 48 h after seeding and plotted the real component for centers of mass for 9 cells demonstrating shifts in metabolic states from OxPhos to glycolytic states (3 cells), from glycolytic to OxPhos (4 cells) and no change (2 cells). The chart was created in Excel using data input from Leica LAS-X software and the color scale-bar with similarly colored circles were generated in Adobe Illustrator. (**d**) In J82 cells treated with cisplatin at IC50, we tracked a cell that shifted quickly from glycolytic (top cyan cell) to an OxPhos state (same cell, yellow and orange) whereas a neighboring cell remained glycolytic (lower cyan cell). Cell colors reflect real metabolic states determined by phasor position relative to the color scale bar in (**a**) and (**b**). (**e**) When analyzed in total from initial treatment to 22.5 h post treatment, the percentage of cells that are OxPhos rises to 42% (red data points) compared with 5% for untreated cells (black data points). (**f**, **g**) J82 cells were cultured in the absence (f) or presence of phenformin (**g**) and analyzed in FLIM phasors, demonstrating a clear shift away from OxPhos (**f**) and toward glycolytic (**g**) metabolism. (**h**) Co-treatment with cisplatin and phenformin produced synergistic reduction in cell count (CDI 0.69, *p* = 0.048). *p < 0.01, calculated using *t* test.

First, FLIM signatures were measured in pure, FACS-sorted SP and NSP cells (Fig. 3a, b). Hoechst dye is necessary for FACS sorting of SP and NSP cells, but it has an emission spectrum that overlaps with NADH fluorescence emission. To eliminate this background noise, NADH FLIM signal was extracted by thresholding out the brightest pixels which corresponded to the nuclear Hoechst dye. The remaining fluorescent signal within the cytoplasm of the J82 cells is relatively photon-limited and therefore needed denoising and amplification of signal-to-noise ratio (SNR). To accomplish this, we applied a recently published filtering method for FLIM phasors, Complex Wavelet Filter^33^. Using this approach, the FLIM phasor distribution of SP cells (Fig. 3a) was distinct from that of NSP cells (Fig. 3b). SP cells had a higher bound/total-NADH ratio (BTNR), 0.85 ± 0.05, as compared to the BTNR of NSP cells, 0.7 ± 0.07, connoting a greater OxPhos metabolic state in SP cells, consistent with our RNAseq, metabolomic, and functional observations. Having assigned differential FLIM signatures to SP and NSP cells, we were able to conduct cell fate tracking experiments for the first time without the constraints of prior Hoechst staining and FACS sorting, as we had originally set out to do. Accordingly, in subsequent experiments where FLIM alone was used to identify the aggressive, drug resistant phenotype, the SP-like cells are termed “OxPhos” cells, as distinct from NSP-like “Glycolytic” cells.

Next, we used FLIM to characterize and track single cell metabolic fates over 48 h (Fig. 3c), using the BTNR cut-off of 0.8 established in earlier experiments to assign OxPhos (> 0.8) versus Glycolytic (< 0.8) phenotype to cells of interest. When randomly examining 4 fields of view at 25× magnification (35 cells total), we found four examples of cells whose metabolic states shifted spontaneously from glycolytic to OxPhos and three other cells that shifted from OxPhos to glycolytic in that time frame (Fig. 3c). This bidirectional change is consistent with our prior plasticity findings^7,12,13^. To assess the effect of drug treatment on cell fates, we treated J82 bladder cancer cells with Cisplatin at IC50 (25 μM) and tracked them in culture for 48 h. In the first 13 h, metabolic FLIM signal was collected at 30-min intervals. During the 5.5–13 h window, we tracked one example of a cell that transitioned from Glycolytic to OxPhos, while its neighbor maintained its initial glycolytic state (Fig. 3d). For the remaining time frame (13–48 h), FLIM imaging intervals were expanded to every hour. After 48 h, cell survival (49% of initial cell count) was consistent with administration of an IC50 dose of Cisplatin, and the OxPhos population as a percentage of total surviving cells climbed to 42% versus 5% for the untreated cells (Fig. 3e). To further confirm that the FLIM signature is not cell line specific, we performed the same experiment in UM-UC-3 (Supplemental Fig. S4a–d) and T24 cells (Supplemental Fig. S4e–h) and observed a similar trend as early as 2 h after cisplatin treatment.

In order to functionally validate the link between the FLIM signature, metabolic state, and cisplatin resistance, we treated cells with phenformin, an inhibitor of mitochondrial complex I and OxPhos metabolism. Phenformin treatment caused a significant shift in the FLIM signature (Fig. 3f–g), away from OxPhos and toward glycolytic metabolism. Consistent with this, phenformin treatment synergistically increased cell death in response to cisplatin (CDI 0.69, *p* = 0.048) (Fig. 3h), suggesting that interference with the rapid transition to OxPhos metabolism reduces cancer cells’ resistance to cisplatin treatment. Similar results were obtained using UM-UC-3 and T24 bladder cancer cells (Supplemental Fig. S4i–j).

## Discussion

Tumor cells have long been known to exhibit unique adaptive metabolic profiles, most notably aerobic glycolysis (Warburg effect)^34^. Recent work has added a new layer of complexity to this model by demonstrating that tumor cells in fact retain functional mitochondria, which play an instrumental role in integrating signals and metabolically adjusting cellular activity in stressful conditions, termed reverse-Warburg or Crabtree effect^35^. Mitochondrial DNA is now recognized as playing key driver roles in cancer progression and adaptability, and upregulated mitochondrial biogenesis and higher OxPhos levels have been reported for different cancer types and cancer stem cells^36^. For example, one recent study of transcriptional classification of IDH wild-type GBM defined a ‘mitochondrial GBM’ that exclusively relied on OxPhos and exhibited marked vulnerability to OxPhos inhibitors^37^. Another recent report showed that mitochondrial ATP powers drug efflux pumps that contribute to cancer cell drug resistance, a mechanism that can be mitigated by treatment with an OxPhos inhibitor^15^. Other studies of tumorigenic, drug resistant cancer stem-like cells have demonstrated distinct metabolic states for these subpopulations^38–41^ with the preponderance of evidence suggesting an OxPhos phenotype. For example, side population cells in lung cancer^42^, sphere-forming and CD133þ cells in glioblastoma^43^ and pancreatic ductal adenocarcinoma (PDAC)^44^, and ROS low quiescent leukemia stem cells^45^ all exhibited preferential OxPhos energy production.

Importantly, while prior studies showed that certain tumor types or cell subpopulations exhibited distinct metabolic states, the manner in which those states arose remained obscure. It might result from canonical selection processes, whereby clonal subpopulations with distinct metabolic features emerged over weeks or months of cell divisions or drug selection. Alternatively, these metabolic distinctions are part of a much more rapid and dynamic phenotypic shift, like the one described recently by our group and by others^7,8,12,13,46,47^.

Here, for the first time to our knowledge, we observe directly that single cancer cells are indeed capable of rapid metabolic shifts over the course of hours, and that these transitions are dramatically amplified with exposure to chemotherapy. Transcriptional profiling of isogenic SP and NSP cells sorted from the same bladder cancer cell line identified the OxPhos pathway as the most highly upregulated in the aggressive, drug resistant SP cells relative to NSP cells. Consistent with this, LCMS comparison of the two cell subpopulations revealed that TCA cycle and oxidative metabolism was upregulated in SP, a finding that was functionally corroborated by Seahorse experiments showing a significantly higher oxygen consumption rate in SP cells. Assigning an OxPhos metabolic signature to SP cells enabled, for the first time, the prospect of tracking the transition to and from this aggressive, drug resistant state in real time.

We sought a systemic technique which would allow us to monitor the metabolic signatures of single cells quantitatively and simultaneously with minimal perturbation. Two-photon FLIM is a powerful non-invasive optical method that yields information on cell metabolism in cells and tissues by correlating the decay profile of endogenous autofluorescent light to a specific biomolecular source^21,30,31^ By localizing and quantifying specific molecular components such as NADH without the need for ectopic tropic labels, it constitutes a powerful tool for tracking in-vivo metabolic changes associated with stem cell differentiation, cancer development and progression, response to chemotherapy, and other biological processes^22–24,48^. Moreover, the cellular and subcellular resolution of FLIM makes it ideal for tracking metabolic transitions of individual cells noninvasively over time.

We therefore performed FLIM analysis on sorted pure SP and NSP cells, and then on unsorted cells in culture over time, both with and without cisplatin treatment. As expected, based on the prior transcriptomic and metabolomic profiling of bulk cells, SP cells exhibited a more OxPhos FLIM signature that was readily distinguishable from the more Glycolytic signature of NSP cells. When we applied this FLIM profile to unperturbed cells (no Hoechst, no FACS sorting) growing in culture, we were able to identify OxPhos cells and Glycolytic cells and then observe them convert from one metabolic state to the other in real time. When cisplatin was administered, the more drug resistant OxPhos cells maintained their OxPhos metabolic FLIM signature, whereas more drug sensitive glycolytic cells transitioned in greater numbers towards a more drug resistant OxPhos state or died from the treatment. Conversely, when the mitochondrial Complex I inhibitor phenformin was applied, the FLIM signature shifted away from OxPhos and toward glycolytic metabolism, with a concomitant reduction in cisplatin resistance. These rapid mitochondrial-driven shifts in metabolic state and drug resistance are not consistent with alterations in nuclear or mitochondrial DNA and are more likely attributable to reversible epigenetic regulation^49^ or post-translational modifications of mitochondrial proteins^49,50^, regulatory mechanisms affecting the electron transport chain that are the subject of ongoing investigation.

Collectively, our findings demonstrate that metabolic plasticity plays a key role in the rapid phenotypic shifts to and from a drug resistant state. Specifically, transition to a mitochondria-mediated OxPhos state can occur stochastically as part of the “bet-hedging” adaptation of cancer cell populations, and also in a more rapid and coordinated manner when drug selection is applied. Metabolic plasticity is a complex process involving multiple oncogenes (e.g. BCL2) and phenotypic shifts like EMT, as well as cross-talk with the tumor microenvironment^46,51^. For example, higher OxPhos in tumor cells has been shown to induce MHC-I expression, rendering cells more sensitive to cytotoxic T lymphocytes (CTL) mediated lysis but more resistant to NK cell-mediated lysis^51,52^.Collectively, these observations underscore the important role played by OxPhos and highlight this metabolic shift as a potential therapeutic target. Concomitant OxPhos inhibition with chemotherapy—as done with phenformin in this study—may curtail cancer cells’ ability to evade toxicity when first exposed. Moreover, the ability to track single cells and groups of cells noninvasively with FLIM as they shift to a more drug resistant metabolic state can be readily applied to a broad array of tumor models to identify, recover, and further characterize cells as they undergo this transition, yielding new mechanistic insights and therapeutic targets to surmount drug resistance.

## Materials and methods

### Key resource table

**Table:** 

Chemicals, drug		
Hoechst 33342 solution 10 mg/ml	ThermoFisher Scientific	Cat# H3570
Cisplatin	Millipore-Sigma	Cat# 232021
[U-^13^C]-labeled glutamine	Cambridge Isotope Laboratories	Cat# CLM-1822-H
[U-^13^C]-labeled glucose	Cambridge Isotope Laboratories	Cat# CLM-1396

### Resource availability

#### Lead contacts

Further information and requests for resources and reagents should be directed to and will be fulfilled by the Lead Contact, Amir Goldkorn (agoldkor@med.usc.edu).

### Material availability

All unique/stable reagents generated in this study will be made available on request.

## Experiment models and subject details

### Cell line

As previously described^7,12^ human bladder cancer cell line J82 (RRID:CVCL_0359) is a generous gift from the laboratory of Dr. Peter Jones. We authenticated the cell line prior to starting the experiments using 9-marker STR profiling (IDEXX BioAnalytics). Interspecies contamination test and mycoplasma PCR evaluation were both negative (IDEXX BioAnalytics). It was routinely maintained in out lab using DMEM and RPMI 1640, respectively (Mediatech, Inc., Manassas, VA) supplemented with 10% heat-inactivated fetal bovine serum (Omega), 1% penicillin (100 units/ml, Invitrogen), and 1% streptomycin (100 μg/ml, Invitrogen) at 37 °C, 5% CO_2_. UM-UC-3 cells (ATCC CRL-1749™) and T24 cells (ATCC HTB-4™) were cultured in DMEM (Invitrogen) supplemented and cultured just as described for J82 cells.

## Methods details

### Measurement of oxygen consumption rate (OCR)

OCR was measured using Seahorse XF Cell Mito Stress Test (Agilent, Santa Clara, CA, USA) at the USC Leonard Davis School of Gerontology Seahorse Core. Briefly, SP and NSP cells were sorted by flow cytometry and seeded at 10^4^ per well in 15 replicates. Assays were initiated by replacing the growth medium with 175 μL XF assay medium (specially formulated, unbuffered Dulbecco's modified Eagle's medium for XF assays; Seahorse Bioscience) supplemented with 2 mM sodium pyruvate and 25 mM glucose, pH 7.4. The cells were kept in a non–CO_2_-incubator for 60 min at 37 °C before placement in the Analyzer. The cells were treated with 1 μM oligomycin; 1 μM tri-fluorocarbonylcyanide phenylhydrazone (FCCP); and 0.5 μM mixture including rotenone and antimycin A according to the instruction. Seahorse XFe Wave Software (Agilent) was applied to analyze the data. All readings were normalized to DNA concentration using Hoechst dye. Average values from 15 replicates were plotted. Student t test were used for statistical analysis.

### Flow cytometry

Hoechst staining and FACS analysis and sorting were conducted as described previously^12,13^. Briefly, J82 cells were trypsinized, counted, and resuspended in prewarmed 10% FBS DMEM media at a concentration of 10^6^/mL. Hoechst 33,342 was added at concentration of 5 μg/mL, incubated for 2 h in 37 °C water bath and gently inverted several times during the course of incubation. Cells were washed and resuspended in ice-cold DMEM media. 7-AAD used to discriminate dead cells was added to the cells at a final concentration of 2 μg/mL. Samples were incubated for at least 5 min on ice before FACS analysis and sorting (FACSAria and FACSLSR-II, BD Biosciences, both equipped with UV lasers).

### Cisplatin treatment

J82, UM-UC-3 cells, or T24 cells were seeded onto a thin bottomed polymer culture dish (μ-Dish 35 mm high wall #1.5 polymer bottom, Ibidi) and incubated at 37 °C, 5% CO_2_ for 2 h before FLIM analysis. After acquiring the initial time point FLIM signature, 25 or 10 μM Cisplatin (Sigma-Aldrich, St Louis, Mo.) was added to the culture. Cell condition was maintained at 37 °C, 5% CO_2_ in the microscope by Tokai-HIT environmental chamber during imaging over 48 h while FLIM signatures were checked at designated time points.

### Phenformin treatment

J82, UM-UC-3 cells, or T24 cells were seeded in an Ibidi black μ-Plate at 0.1 million cells per well seeding density (Cat. No. 82406, Ibidi). After 24 h the cells were treated with phenformin 20 μM (Cat. No. P7045, Sigma Aldrich) and incubated for 72 h before imaging. The untreated controls and Phenformin-treated wells were set up in triplicates.

### Drug resistance assay

J82, UM-UC-3 cells, or T24 cells were seeded in a 6-well plate (0.30 million cells/well), and after 24 h the cells were treated in triplicate with the respective reagents: DMSO vehicle control, cisplatin-10 μM alone (Cat. No. 23120, Sigma Aldrich), phenformin-20 μM alone, or cisplatin + phenformin combination. After 72 h of treatment, the cells were trypsinized and counted using trypan blue exclusion assay. Cell counts were used to calculate a coefficient of drug interaction (CDI) to evaluate synergy, where CDI = [combination/control]/[(cisplatin/control)x(phenformin/control)]; CDI < 1.0 indicates synergy and CDI < 0.7 indicates significant synergy.

### Mass spectrometry-based metabolomics analysis

#### Sample preparation

For flux analysis, culture media was replaced after 24 h by new media containing [U-^13^C]-labeled glucose or glucose, or [U-^13^C]-labeled glutamine (Cambridge Isotope Laboratories). Intracellular metabolite extraction was performed 24 h after adding labeled media. Immediately after FACS, cells were washed on ice with ice-cold 150 mM ammonium acetate (NH4AcO, pH 7.3). After removal of ammonium acetate, cells were re-suspended in − 80 °C cold 80% MeOH, then samples were incubated at − 80 °C for 20 min. Samples were spun down at 4 °C for 5 min at 5 k rpm. The supernatants were transferred into LoBind Eppendorf microfuge tube, and the cell pellets were re-extracted with 200 µl ice-cold 80% MeOH, spun down and the supernatants were combined. Metabolites were dried at room temperature under vacuum and re-suspended in water for LC–MS run. All data were collected from three biological replicates from multiple cell sorting process.

#### Liquid chromatography-mass spectrometry

Samples were analyzed on a Q Exactive Plus hybrid quadrupole-Orbitrap mass spectrometer coupled to an UltiMate 3000 UHPLC system (Thermo Scientific). The mass spectrometer was run in polarity switching mode (+ 3.00 kV/− 2.25 kV) with an m/z window ranging from 65 to 975. Mobile phase A was 5 mM NH4AcO, pH 9.9, and mobile phase B was ACN. Metabolites were separated on a Luna 3 µm NH2 100 Å (150 × 2.0 mm) column (Phenomenex). The flowrate was 300 µl/min, and the gradient was from 15 A to 95% A in 18 min, followed by an isocratic step for 9 min and re-equilibration for 7 min. All samples were run in biological triplicate.

#### Metabolomic data analysis

Metabolites were detected and quantified as area under the curve based on retention time and accurate mass (≤ 5 ppm) using the TraceFinder 3.3 (Thermo Scientific) software. Raw data was corrected for naturally occurring ^13^C abundance. Data was normalized to the sum of total signals in each sample. Pathway maps were made with Cytoscape. For each experiment, the p-value was calculated with a two tailed Student’s *t* test. To evaluate combined significance from independent experiments, *p* values were combined with Fisher’s method and then corrected for multiple hypothesis testing using the Benjamini–Hochberg method.

#### Metabolite set enrichment analysis (MSEA)

Intracellular pool sizes or ^13^C fractional contribution data was ranked based on signed log_10_ FDR q-value comparing SP/NSP populations, and pathway enrichments were calculated using the unweighted statistic in the GSEA java applet.

### RNA sequencing and Gene Set Enrichment Analysis (GSEA)

J82 cells were sorted into SP and NSP in 4 biological replicates. RNA extractions were done using the Direct-zol RNA Microprep (Zymo Research, Irvine, CA, USA), and the sequencing libraries were prepared using the Illumina Stranded Total RNA Prep with Ribo-Zero Plus kit (Illumina, San Diego, CA, USA). Sequencing was performed on an Illumina NextSeq 550 System using a single-end 50-base-pair sequencing targeting 30 million reads per sample. After alignment with hg19 and gencode v19 using STAR v2.7.6a, the differential expression was analyzed from the read counts using limma v3.44.3 in R-4.0.2. Scaled TPM (Transcripts Per Kilobase Million) data were used to construct the heatmap.

To perform GSEA, the gene expression data from RNAseq was ranked by the moderated t-test statistic calculated in limma. Metabolic pathways were downloaded from KEGG, and pathway enrichments were calculated using the unweighted statistic in the GSEA java applet.

### Non de-scanned multiphoton fluorescence lifetime imaging (FLIM)

Fluorescence lifetime images were acquired with a Leica SP8 DIVE FALCON inverted microscope coupled to a tunable infrared laser system (Spectra Physics Insight3X). For image acquisition the following settings were used: image size of 1024 × 1024 pixels and pixel dwell time of 12.6 μs/pixel. FLIM images were acquired from 440 to 475 nm bandwidth on the Leica Non-Descanned hybrid detectors. FLIM data were acquired and analyzed using the Leica FLIM/FCS module. The excitation wavelength was 740 nm with an average power of about 0.6 mW. FLIM data was collected in 7 integrated frame repetitions. We also verified that any autofluorescence due to culture media would not interfere with either the FLIM or the spectral autofluorescent signature of cells.

## FLIM data analysis

Every pixel of the FLIM image was transformed to one pixel in the phasor plot as previously described and reported in detail^21,30,31^. The coordinates g and s in the phasor plot were calculated from the fluorescence-intensity decay of each pixel of the image by using Fourier transformations. Analyses of phasor distributions were performed by cluster identification. Individual cells or regions of interest (ROI) within an image were segmented by hand and the selected image pixels are analyzed within the phasor plot. Segmentation resulted in distributions of pixels and centers of mass were determined for each ROI. Position of the center of mass for an ROI is a measure of the average BTNR for that ROI (Supplemental Fig. S3b).

## Supplementary Information

Supplementary Information.

## Data Availability

The datasets generated during the current study are available from the corresponding author on reasonable request.
