# Supplementary material for: Bladder cancer cells shift rapidly and spontaneously to cisplatin-resistant oxidative phosphorylation that is trackable in real time
Source: Sci Rep. 2022 Apr 1;12:5518. doi: 10.1038/s41598-022-09438-9 (PMC8976067; doi:10.1038/s41598-022-09438-9)
Supplement: Supplementary file 1 — Supplementary Information. [file 41598_2022_9438_MOESM1_ESM.pdf]

Supplemental Information

Bladder cancer cells shift rapidly and spontaneously to cisplatin-resistant oxidative phosphorylation that is trackable in real time

Tong Xu[1§], Jason A Junge[2§], Alireza Delfarah[3], Yi-Tsung Lu[1], Cosimo Arnesano[2], Maheen Iqbal[1], Kevin Delijani[1], Tien-Chan Hsieh[1], Emmanuelle Hodara[1], Hemal H Mehta[4], Pinchas Cohen[4], Nicholas A Graham[3], Scott E Fraser [2], Amir Goldkorn[1*]

[1] Division of Medical Oncology, Department of Internal Medicine, University of Southern California Keck School of Medicine and Norris Comprehensive Cancer Center, Los Angeles, CA 90033

[2] Department of Biomedical Engineering, University of Southern California, Los Angeles, CA 90089, USA; Department of Stem Cell Biology and Regenerative Medicine, University of Southern California, Los Angeles, CA 90033, USA; Translational Imaging Center, University of Southern California, Los Angeles, CA 90089, USA; Molecular and Computational Biology, University of Southern California, Los Angeles, CA 90089, USA.

[3]Mork Family Department of Chemical Engineering and Materials Science, University of Southern California, Los Angeles, CA 90089,

[4]The Leonard Davis School of Gerontology, University of Southern California, Los Angeles, California 90089

[§]T. Xu and J.A. Junge contributed equally to this work.

Corresponding author: Amir Goldkorn, M.D., 1441 Eastlake Avenue, Suite 3440, Los Angeles, CA 90033, agoldkor@med.usc.edu;

Supplemental Figure S1.

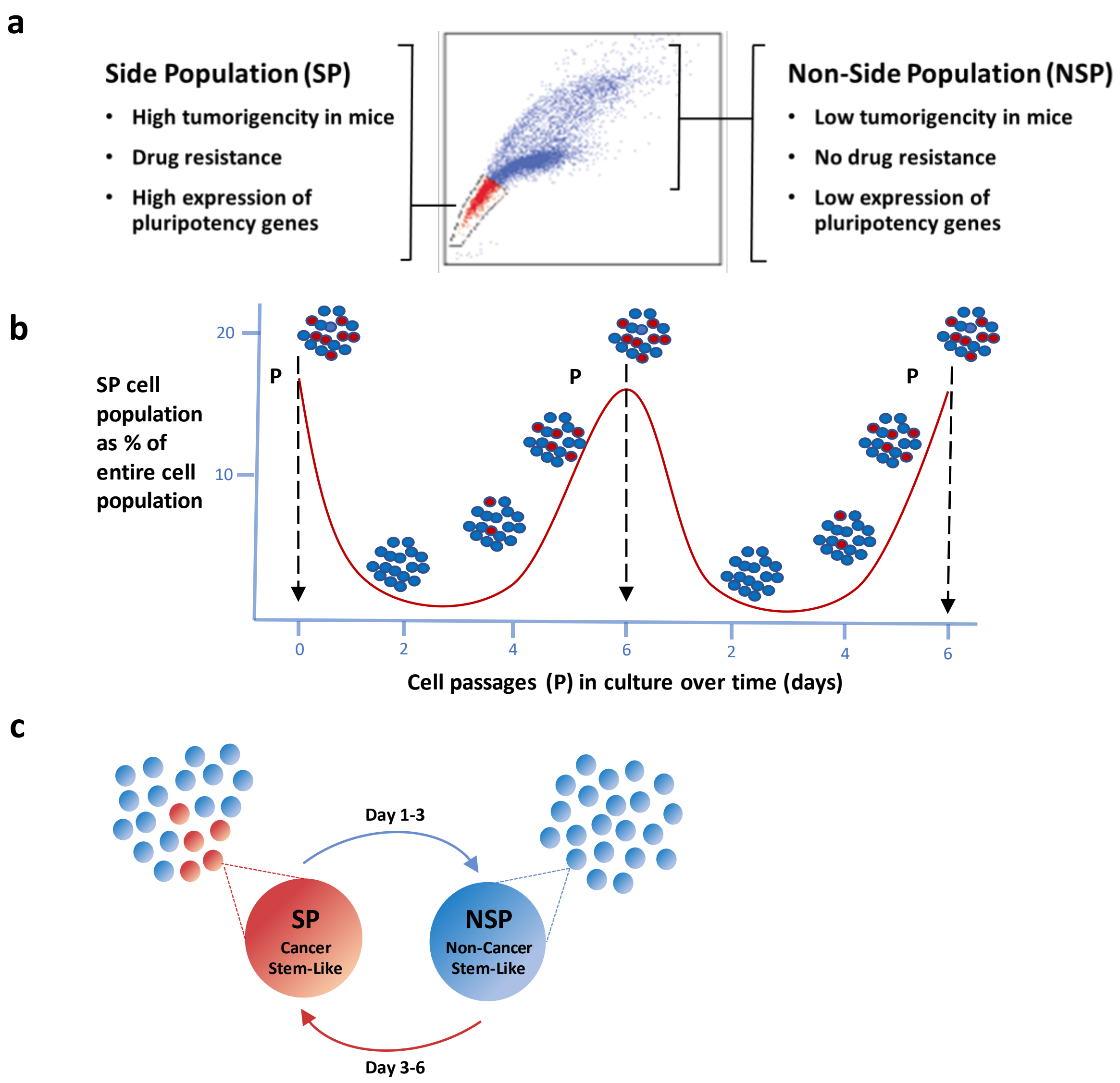

Supplemental figure S1. Plasticity of cancer stem-like subpopulations in bladder cancer cell lines[1]
(a) Two cancer cell states coexist: Hoechst staining and flow cytometry differentiates Side Population (SP: high tumorigenicity, drug resistance, and pluripotency gene expression) from Non-Side Population (NSP: low tumorigenicity, drug resistance, and pluripotency gene expression). (b) Dynamic equilibrium: Cancer stem-like Side Population (SP) fluctuates in size spontaneously and cyclically over serial passages. (c) Phenotypic plasticity: Cancer stem-like Side Population cells differentiate into Non-Side Population Cells, and Non-Side Population Cells convert back into Side Population Cells. Importantly, SP cells cyclically disappear and re-emerge *not* through expansion of existing SP cells, but rather through direct conversion of NSP cells to the SP phenotype. Panel 1c was generated in Microsoft PowerPoint.

Supplemental Figure S2.

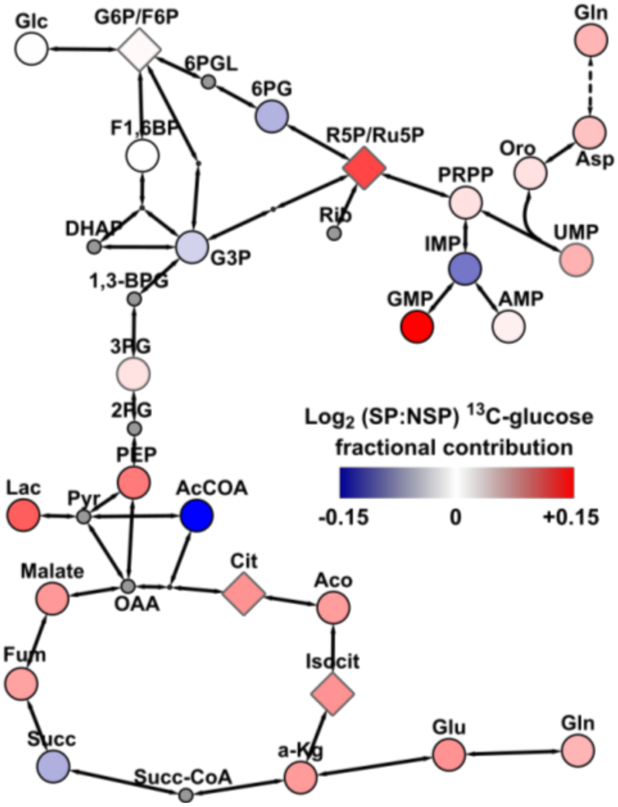

Supplemental Figure S2. Metabolic pathway map representing $\log_2$ fold change of SP/NSP on a color scale for [U-^{13}C]-glucose total fractional contribution. Metabolites that were not measured or had less than 1%fractional contribution are shown as small circles with grey color. Isomers that were not resolved by LC-MS are shown as diamonds. Generated in Cytoscape: version 3.5.1. https://cytoscape.org/roadmap.html

Supplemental Figure S3.

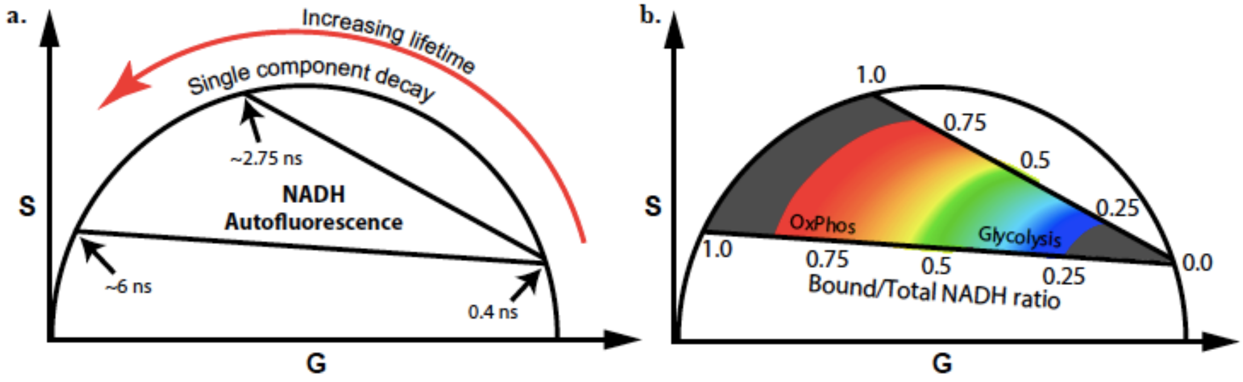

Supplemental Figure S3. Phasor overview and roadmap. Digman et al.[2] describe in detail how the phasor is calculated, and the phasor roadmap (a) shows lifetime trends within the FLIM phasor. The semi-circle marks where single-component exponential decays are plotted. In buffer, 100% unbound NADH has a single-component exponential decay of 0.4 ns and localizes very specifically to the lower right side of the semi-circle. NADH, in vivo, has a complex FLIM signal that is made of many components[3] which typically plot within the wedge-shaped region in the phasor (a and b). The rainbow scale bar (b) is an example of how we maintain a consistent relationship between Bound/Total NADH ratiometric measurements (fractional distances along cords) and our displayed colors.

Supplemental Figure S4

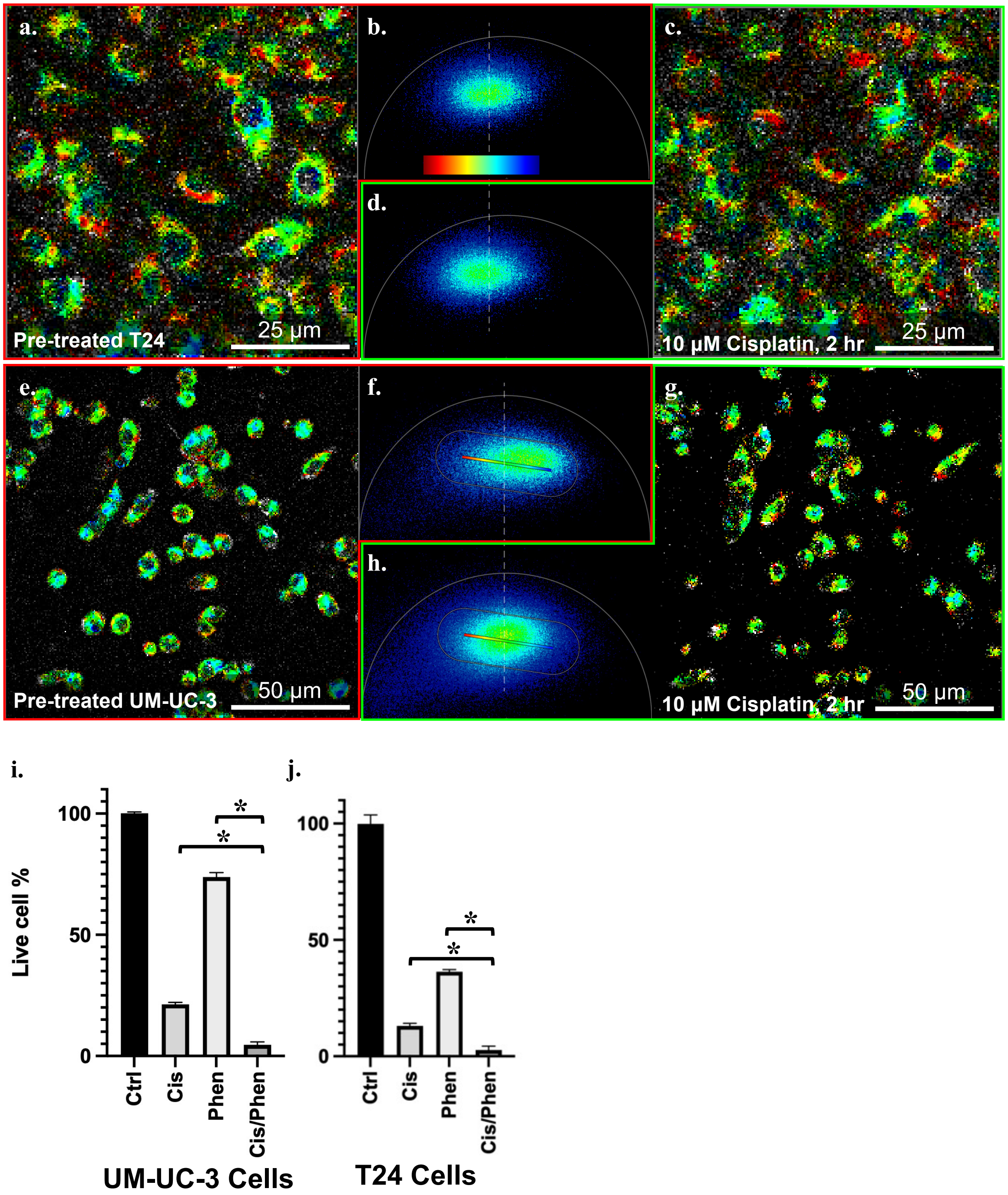

Supplemental Figure S4. FLIM and drug resistance assays for UM-UC-3 and T24 bladder cancer cells. FLIM phasors were used to analyze T24 (a-d) and UM-UC-3 cells (e-h) both preceeding and 2 hours after cisplatin treatment. Pre-treatment, both cell lines have phasor distributions which describe shifts from more glycolytic toward more Oxphos metabolic states (T24, 64% bound NADH in (b) to 68% bound NADH in (d)) (UM-UC-3, 40% bound NADH in (f) to 50% bound NADH in (h)) after cisplatin treatment. The lines of demarcation between panels b&d and f&h show the extent of the shift of their phasor positions and the lookup tables for corresponding phasors are presented in b for b&d and in each phasor for f&h. T24, N=2 (74 cells); UM-UC-3, N=3 (142 cells). Co-treatment with cisplatin and phenformin in UM-UC-3 (i) and T24 (j) cells produced synergistic reduction in cell count (UM-UC-3,CDI 0.3; T24, CDI 0.57). *p <0.01, calculated using t-test.

References

1. Xu, T.*, et al.* Epigenetic plasticity potentiates a rapid cyclical shift to and from an aggressive cancer phenotype. *Int J Cancer* **146**, 3065-3076 (2020).
2. Digman, M.A., Caiolfa, V.R., Zamai, M. & Gratton, E. The phasor approach to fluorescence lifetime imaging analysis. *Biophys J* **94**, L14-16 (2008).
3. Leben R, Köhler M, Radbruch H, Hauser AE, Niesner RA. Systematic Enzyme Mapping of Cellular Metabolism by Phasor-Analyzed Label-Free NAD(P)H Fluorescence Lifetime Imaging. Int J Mol Sci. **22**, (2019)

Supplemental table S1.

Raw metabolite data

Metabolite	Sample	Isotopomer	Percentage Mean	Percentage Standard Deviation
CITRATE/ISOCITRATE	NSP	M0	4.13E-01	2.17E-03
CITRATE/ISOCITRATE	NSP	M1	3.40E-02	1.52E-04
CITRATE/ISOCITRATE	NSP	M2	5.50E-02	5.46E-04
CITRATE/ISOCITRATE	NSP	M3	4.57E-02	4.76E-04
CITRATE/ISOCITRATE	NSP	M4	2.71E-01	7.75E-04
CITRATE/ISOCITRATE	NSP	M5	1.76E-01	5.35E-04
CITRATE/ISOCITRATE	NSP	M6	4.98E-03	1.34E-04
CITRATE/ISOCITRATE	SP	M0	3.98E-01	1.99E-03
CITRATE/ISOCITRATE	SP	M1	3.39E-02	2.80E-04
CITRATE/ISOCITRATE	SP	M2	5.69E-02	5.23E-04
CITRATE/ISOCITRATE	SP	M3	4.14E-02	1.04E-04
CITRATE/ISOCITRATE	SP	M4	3.20E-01	6.26E-04
CITRATE/ISOCITRATE	SP	M5	1.46E-01	1.11E-03
CITRATE/ISOCITRATE	SP	M6	3.64E-03	1.00E-04
ACONITATE	NSP	M0	4.06E-01	5.02E-03
ACONITATE	NSP	M1	2.92E-02	2.96E-03
ACONITATE	NSP	M2	5.19E-02	1.15E-03
ACONITATE	NSP	M3	4.36E-02	6.35E-04
ACONITATE	NSP	M4	2.87E-01	9.40E-04
ACONITATE	NSP	M5	1.80E-01	1.72E-03
ACONITATE	NSP	M6	2.08E-03	6.56E-04
ACONITATE	SP	M0	3.90E-01	1.67E-03
ACONITATE	SP	M1	2.83E-02	2.97E-03
ACONITATE	SP	M2	5.31E-02	2.62E-03
ACONITATE	SP	M3	3.89E-02	1.78E-03
ACONITATE	SP	M4	3.40E-01	2.54E-03
ACONITATE	SP	M5	1.49E-01	7.62E-04
ACONITATE	SP	M6	1.15E-03	5.24E-04
